# The relationship between primary healthcare providers and their external supervisors in Rwanda

**DOI:** 10.4102/phcfm.v9i1.1508

**Published:** 2017-11-01

**Authors:** Michael Schriver, Vincent K. Cubaka, Laetitia Nyirazinyoye, Sylvere Itangishaka, Per Kallestrup

**Affiliations:** 1 Centre for Global Health, Department of Public Health, Aarhus University, Denmark; 2 Aarhus University Hospital, Denmark; 3 School of Medicine and Pharmacy, College of Medicine and Health Sciences, University of Rwanda, Rwanda; 4 School of Public Health, College of Medicine and Health Sciences, University of Rwanda, Rwanda

## Abstract

**Background:**

External supervision of Rwandan primary healthcare facilities unfolds as an interaction between supervisors and healthcare providers. Their relationship has not been thoroughly studied in Rwanda, and rarely in Africa.

**Aim:**

To explore perceived characteristics and effects of the relationship between providers in public primary healthcare facilities and their external supervisors in Rwanda.

**Setting:**

We conducted three focus group discussions with primary healthcare providers (*n* = 16), three with external supervisors (*n* = 15) and one mixed (*n* = 5).

**Methods:**

Focus groups were facilitated under low-moderator involvement. Findings were extracted thematically and discussed with participating and non-participating providers and supervisors.

**Results:**

While external supervision is intended as a source of motivation and professional development in addition to its managerial purpose, it appeared linked to excessive evaluation anxiety among Rwandan primary healthcare providers. Supervisors related this mainly to inescapable evaluations within performance-based financing, whereas providers additionally related it to communication problems.

**Conclusion:**

External supervision appeared driven by systematic performance evaluations, which may prompt a strongly asymmetric supervisory power relation and challenge intentions to explore providers’ experienced work problems. There is a risk that this may harm provider motivation, calling for careful attention to factors that influence the supervisory relationship. It is a dilemma that providers most in need of supervision to improve performance may be most unlikely to benefit from it. This study reveals a need for provider-oriented supportive supervision including constructive attention on providers who have performance difficulties, effective relationship building and communication, objective and diligent evaluation and two-way feedback channels.

## Introduction

In sub-Saharan Africa, supervision of primary healthcare facilities is widely practised as part of service management and ensuring quality of care.^1,2,3,4,5^ The relationship between the person (or team) providing supervision and the person (or team) receiving it influences its quality. The supervisory relationship has been described as the single most important determinant of supervision outcome, more so than supervision methods.^6^

A review on supportive forms of supervision in sub-Saharan Africa described its success as relying on the formation of trusting, collaborative supervisory relationships characterised by open, two-way communication.^3^ In contrast, abusive supervisory relationships discourage supervisees and may harm performance.^7,8^ It has been suggested that even supportive supervision forms will not be successful, unless there is a good understanding of the human interactions involved.^5^ Meaningful work relations, including engaging in trusting professional relationships, has been described as a basic human need that determines work motivation.^9^

Power in the supervisory relationship – here referring to the ability of supervisors to influence a change in providers’ practice – has long been explored in the management sciences.^10,11,12,13^ The forms of power applied by a supervisor will shape the supervisory relationship. In French and Raven’s classical model, power may be exerted through a mutually accepted right of the supervisor to require certain tasks of a health provider.^13^ This is legitimised by the supervisor’s professional and hierarchical position.

Other relevant forms of managerial power are reward and punishment.^13^ These are common within managerial supervision systems in sub-Saharan Africa^1,2,4,14^ and may be determined through performance evaluations.^9^ Evaluation has been described as the main manifestation of the supervisory power differential^15^ and is a known source of tension and anxiety.^16,17,18^ Studies on supervision in psychotherapy practice found strong yet complicated interactions between the practice of performance evaluation and quality of the supervisory relationship.^19^ Supervisors carrying dual roles of performance evaluator and clinical supporter may find the trust from supervisees threatened if evaluations take place in the absence of basic mutual understanding.^20^

Supervisors’ positional power over providers usually implies a special responsibility for promoting effective supervisory relationships.^15,19^ Inasmuch as the mentioned forms of power do not take account of needs and opinions of providers, they may be described as restrictive,^21^ which implies a somewhat controlled form of work motivation.^9^ To the extent they are in accordance with knowledge, interests and needs of the provider, they may be described as promotive,^21^ indicating rather a self-determined behaviour under autonomous motivation.^9^

### Context

There are around 495 health centres (HCs) in Rwanda, and they deal with more than 90% of all outpatient visits in the health system.^22,23^ HCs only offer primary healthcare services and have no medical doctors. More than 90% of HC nurses have a basic secondary school–based nursing degree (known as an A2 degree).^24^

In Rwanda, a team of external supervisors in each of 39 district hospitals conduct supervision visits to HCs in that district.^25^ The external supervisors are part of a district health management team. They work within the health management information system and have explicit supervisory functions. A supervisor is usually responsible for supervision within a certain domain such as HIV and TB, laboratory services, maternal and child care, general outpatient clinic services, etc. Depending on the number of HCs under a district hospital, this may in some districts require supervisors to work full time with supervision, including daily field visits sometimes to distant HCs. The size of a supervision team varies according to the number of HCs supervised under the hospital, ranging from as low as 4 to as many as 23 HCs. Anecdotally, supervisors are typically clinically experienced nurses with a higher nursing degree (a so-called A1 or A0 degree) and rarely trained specifically in facilitation of learning or interpersonal communication.

External supervision of HCs includes two categories of supervision visits, in practice often combined, conducted by the same team of supervisors: (1) evaluative and (2) formative.^14^ Evaluative supervision predominates, not least because of a monthly frequency for some tasks. It involves performance marking of indicators of specific services (not of individual providers) to determine reward size to HCs in performance-based financing (PBF). This may be distributed as salary bonuses among providers, as determined by the HC manager.^14,26^ Formative or technical supervision visits are expected yet unelaborated in official documents on health system strategy and monitoring.^27,28^ It emerges as a distinct, intended practice aiming to build staff capacity in various services, but problematised by providers and supervisors as missing or insufficient.^14^

Supervisors are not paired with individual providers and often meet new supervisory counterparts as providers may shift tasks.^29^ Whatever the supervision frequency and familiarity between actors, external supervision of HCs unfolds in essence as a complex interaction between supervisors and providers, whose relationship has not been thoroughly studied in Rwanda, and rarely on the African continent.

This study aimed to explore key characteristics of the relationship between healthcare providers in Rwandan HCs and their external supervisors from the district hospital, with particular focus on exploring major challenges for building effective supervisory relationships and potential avenues to address these. The paper represents the second of two papers using the same overall set of data, where the first paper^14^ focused on the relationship between evaluative and formative supervision functions.

## Method

This is a qualitative study. While we do not claim allegiance with a particular qualitative research tradition, this study was inspired by principles from grounded theory, particularly by using data to propose a theoretical model for improving the supervisory relationship.

### Sampling

The data consist of three focus group discussions (FGDs) with supervisors, three FGDs with providers and one with a mixed group, in total 15 supervisors and 16 providers. All FGDs were conducted in the period October 2013 – January 2014.

We purposefully considered the number of HCs in a district for our sampling because this may influence the number of external supervisors in a team and how much time the supervision team spends on field visits to HCs, which we assumed could impact the supervision experience among supervisors and providers. We therefore included a supervision team from a district with a high number of HCs, a team with few HCs and a team from a district with an average number of HCs. To optimise the chance of retrieving precise recollections, we asked providers from the HC most recently supervised in each of these three districts to participate in a provider discussion. For the same reason, we excluded providers who had not experienced supervision in the past 6 months. HC managers were excluded from the provider FGDs with the intention to set a discussion among peers and reduce social desirability bias. FGDs took place in undisturbed rooms at participants’ main work facility, and participants were offered a drink.

To further deepen the discussion of solutions to challenges in the supervisory relationship, two supervisors and three providers who participated constructively in a FGD also took part in a mixed seventh FGD (see Data collection methods). This was held in a private room at a restaurant.

### Data collection methods

We observed a humorous atmosphere when engaging with providers or supervisors in separate groups, and none so on encounters bringing them together. This indicated warm collegial bonds within rather than between these professional groups. This was one reason to use FGDs within groups of peer colleagues to explore the potentially sensitive issue of how one group of professionals relates to another. Further reasons are provided elsewhere.^14^

All FGDs were conducted with minimal moderator involvement to minimise participants’ attention on the moderator and maximise participants’ attention towards each other.^14,30,31,32^ Thus, participants were facing each other, and the moderator was seated in the background mostly remaining passive and silent, only contributing to steer a discussion back to the topic at hand, clarify topics, probe a relevant discussion if needed or suggest an opinion from relatively silent participants. The moderator was a local social scientist (SI) with experience in conducting group discussions. We presented participants with eight discussion topics directly or indirectly related to the supervisory relationship: (1) Supervision experiences in general, (2) Aims of supervision, (3) Positive experiences, (4) Negative experiences, (5) Supervisors’/supervisees’ (the other’s) behaviour, (6) Supervisors’/supervisees’ (the other’s) view of supervisees/supervisors, (7) Training vs inspection and (8) Recommendations. Some of these (topics 1, 3, 4, and 8) were directly relevant both to this study and the related study,^14^ some mainly to this study (topics 5 and 6) and some (topics 2 and 7) mainly to the related study.^14^

Participants in the mixed FGD were presented with excerpts from previous FGDs about challenges in the supervisory relationship and asked to discuss these. The aim was to gain further nuances and to challenge robustness of the findings of those FGDs, and to retrieve a balanced, two-sided discussion on potential approaches to challenges in the supervisory relationship.

In total, 11 h of effective discussion were audio recorded in Kinyarwanda, transcribed verbatim and translated into English by a professional interpreter. Both transcription and translation were control checked sentence by sentence by the moderator.

### Data analysis

We applied the framework approach for data analysis.^33^ Four coders familiarised themselves with the transcripts and open-coded them paragraph by paragraph. Over multiple meetings, coders discussed and agreed on major code categories for each FGD separately, and subsequently harmonised these to attain one final thematic index covering all FGDs. Two researchers used this to index the material, and intercoder agreement was compared paragraph by paragraph to produce a single harmonised coding of transcripts. One researcher read charted compilations of the indexed material as well as the full material several times, and over discussions among co-authors seven main themes suitable for presentation of findings pertaining to the study aim were determined among several in the thematic index. The software MAXQDA11.2 was used for this process including charting the content of emerging themes.^14,33,34^

To test transferability, our findings were presented at three different meetings (latest in October 2016) to a total of 8 providers and 13 supervisors (among whom 4 had participated in FGDs) for critique and discussion to further refine analysis and conclusions. This did not lead to any significant changes and is considered an important contribution to trustworthiness of the findings.

### Ethical considerations

This study was approved by the Faculty of Medicine Research Ethics Committee at the National University of Rwanda (Review Approval Notice N0 15/FoMREC/2013). Signed informed consent was obtained from all study participants prior to participation. The mixed FGD put together three providers and two supervisors from separate districts to avoid influencing or harming existing supervisory relationships.

## Results

Characteristics of the FGDs and their participants are shown in Table 1 and also presented elsewhere.^14^

**TABLE 1:** Focus group discussions characteristics.

Characteristics	FGD 1	FGD 2	FGD 3	FGD 4	FGD 5	FGD 6	Total
No. of participants	4	4	7	5	6	5	31
Supervisors or providers	Supervisors	Supervisors	Supervisors	Providers	Providers	Providers	15 Supervisors 16 Providers
Age range	30–46	32–56	29–50	31–42	27–41	29–40	27–56
Gender							
Male	2	4	5	0	2	2	15
Female	2	0	2	5	4	3	16
Highest degree							
A2	0	0	1	3	3	4	11
A1	2	2	1	2	3	0	10
A0	2	1	4	0	0	1	8
Other	0	1	1	0	0	0	2
Years since first health prof. graduation range	3–24	5–32	4–20	7–17	4–7	6–11	3–32
No. who had clinical training in past year	Yes: 1 No: 3	Yes: 1 No: 3	Yes: 2 No: 5	Yes: 0 No: 5	Yes: 0 No: 6	Yes: 4 No: 1	Yes: 8 No: 23
Years as supervisor range	2–8	2–10	1–10	-	-	-	1–10

A2, Basic nursing certificate attained during secondary school; A1, Full nursing degree; A0, Bachelor degree.

FGD, focus group discussion.

As the table shows, among the 16 participating providers 12 were female, 13 above 30 years and all had worked more than 4 years as a nurse. Ten had only the lowest A2 nursing degree taken as part of secondary school, while five had an A1 full nursing degree and one the advanced A0 bachelor degree. Among the 15 supervisors, 11 were male, 14 above 30 years and experience as a supervisor ranged from 1 to 10 years.

Concerning participants’ contributions, providers contributed 43% of the total number of characters in the transcripts and supervisors 57%. Individual contributions varied from accounting for a minimum of 1% to a maximum of 7% of all characters, with an average of 3% per provider and 4% per supervisor.

We describe our findings under seven main themes pertaining to the supervisory relationship that emerged from inductive analysis: Power relation; provider competence; emotions in supervision; communication problems; trust in supervisors; coping and reacting; and building the relationship. The term ‘s/he’ and ‘him/her’ within quotes represents a translation of the genderless subject found in the Kinyarwanda language.

### Power relation

The power relation between supervisors and providers was discussed in all FGDs. An essential form of power available to supervisors is related to PBF rewards, which require regular performance marking aimed to regulate the behaviour of providers. Its flip side is a sense of punishment or conflict when marks are low or rewards lack, as indicated by a supervisor in FGD3:

‘When it comes to the sense of evaluation, conflicts arise. It is a conflict of interest because it involves marks between brackets. It becomes a punishment.’ (S15, male, A0 degree)

Disciplinary action against providers was another power source in the potential form of punishment, and something supervisors in general found important. Some felt it was problematic that this power decreased in recent years. For instance, supervisors no longer had the power to suggest or carry through a firing of individual HC staff.

Providers shared stories of supervisors’ inappropriate use of power or active display of a superior hierarchical position. In FGD4, this was often referred to as a ‘superiority complex’:

‘They [*supervisors*] always want to show they are on the above position, treading on you. Therefore, you feel you have to tremble and be afraid.’ (P4, female, A2 degree)

‘That’s the way they normally behave. They often have a superiority complex [*All agreeing*].’ (P1, female, A2 degree)

Sometimes supervisors were likened to police or to businessmen who would treat providers like domestic workers. Supervisors themselves acknowledged that an inappropriate use of power could take place in which supervisors imposed their views, and providers might not get a proper opportunity to talk. Some supervisors showed understanding for the feeling of inferiority among providers by comparing with their own experiences of being supervised.

There were no attempts to justify an inappropriately authoritative behaviour among supervisors. Some providers saw it as a sign of supervisor insecurity, as this dialogue in FGD6:

‘You may also have [*show*] all s/he is asking you and get low marks because s/he wants to keep confidence for it is not her/his domain.’ (P13, female, A2 degree)

‘Therefore, if those who come are not in your domain nor have a more advanced level … they try to complicate things … to show you that they know more than you.’ (P14, female, A2 degree)

### Provider competence

Here, we present perceptions concerning the competence of primary healthcare providers, as most other headings relate to the competence and behaviours of supervisors. Supervisors generally commended the work of providers and acknowledged their stressful working conditions, including shortage of staff. At the same time, some supervisors spoke in generalising or derogatory terms about HC nurses’ competence level, such as a supervisor in FGD1:

‘We can call these employees [*HC staff*] “second hand employees” or “second category employees”, because those who are in the first category are able to come and work at the hospital.’ (S2, male, A0 degree)

Several supervisors described providers in terms essentially separating them in to those with high and low performance, as in FGD1:

‘Those who show interest [*in supervision*] as he said, are those who generally try to do everything as well as they can. Those other ones who need to be reminded are those ones who generally don’t produce quality work.’ (S4, female, A1 degree)

All supervisor discussions described providers who failed to implement changes as recommended or appeared uninterested in supervision. This was often perceived as a behavioural problem, sometimes termed *resistance to change*. Some supervisors showed frustrations with encountering such resistance. Multiple paragraphs indicated the dilemma that providers with performance difficulties could attempt to dodge supervision, such as a supervisor in FGD1:

‘Those [*providers*] who are disorganized are the ones who avoid anybody who would train them on how they should do their work.’ (S4, female, A1 degree)

Supervisors did not give clear explanations for resistance or avoidance among providers. Many supervisors would involve HC managers when providers had problems to perform well. Another approach was to carefully reflect on how to help, as expressed by a supervisor in FGD2:

‘When s/he who has some weaknesses doesn’t want to move from one point to another … we should look for a strategy to help them.’ (S6, male, A0 degree)

Some providers would acknowledge the problem of failure to change their practice, and some related it to a lack of self-or peer supervision. A humble and supportive attitude of the supervisor appeared particularly important when dealing with providers who have performance difficulties. This indicated that resistance to change may reflect an unfit support, which may determine the outcome of supervision, as this nurse in FGD5 expressed:

‘Sometimes the supervisor comes in a good mood, talking to you friendly. … And even if there may be a mistake, s/he corrects you calmly. … Next time, s/he finds that you have adjusted it because s/he had come humbly and with good temper.’ (P8, female, A2 degree)

### Emotions

Emotions shape and are triggered by interpersonal relationships. Although discussion topics did not specifically ask about emotions, thematic codings of emotions (including fear, shame, embarrassment, humility, anger, frustration, etc.) were among the most utilised. Fear emerged as the most frequently described emotion, in all cases supervisees’ fear of supervision/supervisors. Several providers described their fear with classical symptoms of anxiety or even panic, such as excessive sweating, sudden urge to go to the toilet or palpitations upon seeing the supervisors or their car. It was indicated that the supervisor–provider relationship is one between individuals, and fear was sometimes related to an individual supervisor.

Supervisors found that dealing with supervisees’ fear of supervision was a natural part of their work. They could relate to this fear by thinking about their own experiences of being supervised, as in FGD2:

‘It doesn’t happen at health centres only. Even if he [*a national supervisor*] comes to give us some advice, we first feel discomfort. … That fear is in the human nature [*other participants repeat agreeingly*].’ (S8, male, A1 degree)

Supervisors and providers generally related the fear to evaluations and performance marks. A supervisor in FGD2 said:

‘You arrive there and they feel as if the universe has fallen on them [*afraid and worried*] … they feel like you are always going to give them marks.’ (S7, male, degree not reported)

Both supervisors and providers questioned the capacity to learn under fear and mentioned that fear can harm performance, as a provider in FGD6:

‘You can never feel secure when you are under supervision. By just learning that it is coming, you … you can even drop down what you have because of fear, [*laughs*]. … You lose marks! Why? Because of fear.’ (P14, female, A2 degree)

A provider in FGD6 expressed that one could start forgetting because of fear:

‘When you hear that they [*supervisors*] have come, you can even lose your mind and forget where you have put the filing cabinet. … You do not feel they are coming to help you but rather you feel anxious.’ (P16, female, A0 degree)

Other emotions directly or indirectly related to supervision were anger and rage, and some described situations where providers and supervisors had been in harsh confrontations, almost fights. Also, supervisors found that providers often felt embarrassed during their interaction. A supervisor in FGD1 thought that especially providers with performance difficulties felt ashamed:

‘Those who have a low level of knowledge … feel ashamed. … Maybe s/he thinks: “Perhaps s/he [*supervisor*] has discovered that I am stupid or that I have little competency and then s/he has come for challenging me and reporting me to my superiors”.’ (S2, male, A0 degree)

### Communication style

Supervisors and providers consistently described supervisors’ communication style as essential for the supervisory relationship. A provider in FGD4 described how rude interpersonal communication made her feel:

‘S/he [*supervisor*] added: “Anyway, on the asphalt road [*health center is near an asphalt road*], you are all the same like this.” … My God, I felt sad.’ (P4, female, A2 degree)

Providers expressed that the damage of a derogatory or rude communication style was not easily mended even if supervisors provided good advice. Rude communication may leave permanent negative memories, as a provider described in the mixed FGD:

‘It was my first time to work in that service and they came to supervise. … S/he told my boss “why have you appointed that ignorant [*person*] there?”. That thing has never got out of my mind until this hour.’ (P3, female, A1 degree)

Rude communication was especially linked to the discussion of mistakes, as this provider in FGD5 explained:

‘Maybe s/he [*supervisor*] finds out that the same mistake s/he had seen you with last time still exists. Instead of looking for how to adjust it together, it becomes the reason to say that you do nothing in the department, … you are useless there.’ (P7, female, A2 degree)

In FGD3, a supervisor suggested performance evaluations make communication prone to conflicts:

‘Staff of health centres views us as people who have come to create conflicts … because when s/he gets few marks in evaluation, s/he says “it’s going to affect me in PBF. … Now I am not going to get on well with my boss”.’ (S14, male, degree not reported)

Another type of negative communication was related to gender. This issue was discussed at length in FGD4, such as a provider stating:

‘They [*supervisors*] like saying “those women”. … For me, I think they should avoid it.’ (P2, female, A1 degree)

This was also discussed in the mixed FGD where supervisors also found such expressions inappropriate.

Some providers explained that a negative communication experience with a supervisor could persist as anger or emotional discomfort during their clinical duties, unintentionally harming their interaction with patients.

### Trust in supervisors

Issues related to supervisees’ trust in supervisors were mentioned in all FGDs, and trust was found essential for successful supervision including disclosure of weaknesses, as indicated by a supervisor in FGD3:

‘Supervision should be done in such a way that s/he [*provider*] can show you where s/he has weaknesses and even orienting you saying “I have a weakness here, what can you do to help me?” However, instead of doing that, s/he hides something from you.’ (S14, male, degree not reported)

Providers accepted that supervisors had to report to HC managers when attitudes or competences were considered inappropriate. However, several providers complained this was done in improper ways which could harm the trust, such as a provider in FGD4:

‘There were some things which were not well completed. Then, s/he [*supervisor*] thereafter told the chief [*HC manager*] … “What does s/he do in that service?” Yet, I had been working in that service for two years, and s/he talked to my employer without telling me anything. That shows s/he held me up to ridicule without even coming for training me.’ (P3, female, A1 degree)

In FGD4, another provider told a story about public embarrassment:

‘If s/he [*supervisor*] talks about you publicly, … you directly become discouraged up to the point that you feel you would hide yourself under this table…. Imagine 13 health centres [*at a coordination meeting*] with 3 persons per health center, and s/he [*supervisor*] makes you stand up…. Something you were discussing as two people, s/he directly raises the point among all the participants.’ (P2, female, A1 degree)

Some providers felt mistrusted or under suspicion, as in FGD6:

‘If necessary s/he [*supervisor*] says to you, “Why do I see that this signature is not his/her?”. … To make you feel afraid.’ (P14, female, A2 degree)

Other providers expressed the opposite complaint that supervisors sometimes trusted providers too much. This indicated to them that supervision could be conducted in a superficial way.

### Coping and reacting

A number of coping methods and reactions were demonstrated in response to the challenges above. Some providers felt unable to speak during supervision and simply remained silent. Avoidance of supervision, as mentioned, appeared common, here described in a dialogue of two supervisors in FGD1:

‘They [*providers*] usually tend to hide themselves. Upon sight of the car and upon recognition of people who come in it, each one tries to hide him/herself.’ (S4, female, A1 degree)

‘Few are those who are happy about it [*supervision*]. … They are sort of disturbed, … afraid of something which makes some of them tend to go and hide. We see it, we really agree on that fact that some of them go and hide themselves.’ (S1, male, A0 degree)

Several providers showed a sense of apathy when describing a negative supervision experience. A provider in FGD4 showed a somewhat fatalistic attitude when explaining how reproachful words may ignite conflict:

‘Supervisors themselves come and tell us like “It’s known you at [*Name of HC*] have become impossible to manage”. You cannot quarrel with him/her; you only say [*think*] “That’s ok, take it as you already have it in your mind”. You do not tell them anything with humiliation/humility until God will give the final answer.’ (P4, female, A2 degree)

Supervisors noticed the apathy as well, and saw HC managers as an influential actor, as in FGD1:

‘There are also those who don’t mind anything. It is as if they could say to supervisors “Do your job quickly and let us do our usual businesses”. … This happens mostly to people who are unhappy about their leadership or leaders, they are the ones who think that supervisors just come to disturb them or make them waste their time.’ (S2, male, A0 degree)

Some supervisors described a more externalised coping strategy, such as providers trying to influence evaluations through inappropriate negotiations or persuasions. This could be by asking or begging for better marks or a form of bribing such as offering a beer at bar. Some providers believed supervisors expected this. In the mixed FGD, a provider described it could be by using gender attributes:

‘Another colleague … told me “you do not know how to talk to him!”. S/he said, “you get close to him, smile to him and tell him ‘forgive me’”. He is a man – and then he gives you all your marks [*best marks in PBF*].’ (P1, female, A2 degree)

### Building the relationship

All FGDs had suggestions about how the supervisory relationship may be improved. Respectful and friendly forms of communication were generally found essential. Some providers explained that pleasant communication and a humble attitude of the supervisor would compensate the negative experience of a low performance mark, changing it instead into a constructive experience.

Several providers suggested training of supervisors in interpersonal skills is needed. Supervisors also found training relevant. Yet, a supervisor in the mixed FGD thought training in communication might not solve interpersonal problems related to attitudes or values of supervisors:

‘It’s about conscience of everyone. … Training itself is not enough. … In fact, when you do not put your conscience on it, you feel that it [*supervision*] does not mean anything even. You notice that it has become a game rather than something formative.’ (S7, male, degree not reported)

Providers too talked about changes in attitude and not simply communication skills, as in FGD5:

‘Supervisors should change: they should come with disposal for mutual understanding with those whom they supervise, and with humility so that these [*supervisees*] feel comfortable with them and vice versa. This will facilitate teaching and correcting each other, as well as mutual empowerment.’ (P8, female, A2 degree)

Several said providers should be trained in what to expect from formative and evaluative supervision visits. Further, providers expressed a need for a channel of feedback to supervisors as in FGD4:

‘What I suggest is that people [*supervisors and providers*] should sit and have a discussion whereby they [*supervisors*] ask us “Do you think that we do the supervision well?” … in order that there isn’t any favoritism.’ (P3, female, A1 degree)

Others suggested such feedback be in a formalised appraisal in which providers could evaluate supervisors according to their behaviour. Providers thought feedback to supervisors could be facilitated by help from both supervision and HC managers.

## Discussion

Supervisors and providers generally considered supervision highly important. Critique of the supervisory relationship did not appear to question the value of supervision as such, but was rather an input to strengthen it.

### Improving a poor supervisory relationship

**FIGURE 1:**
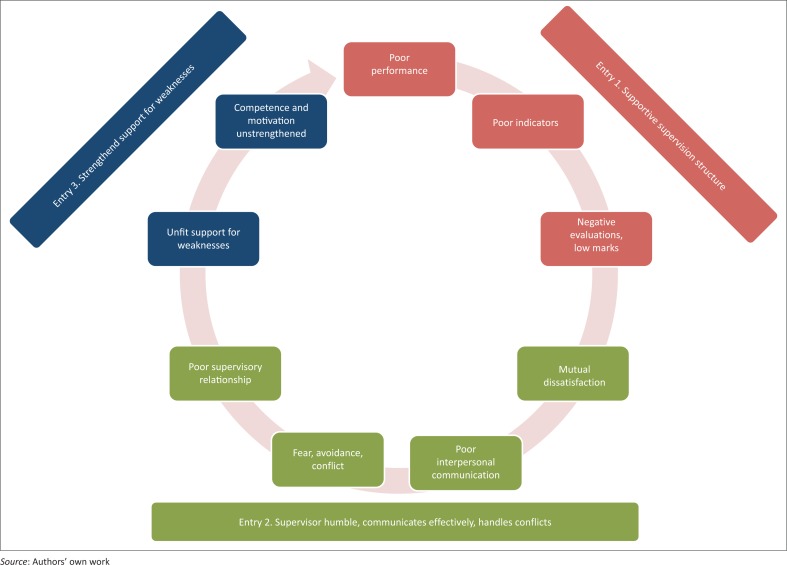
Entries for improvement in a vicious cycle of poor performance.

Drawing on Weberian theory of social action, our data are used to propose an ideal type model of how poor supervisory relationships develop and may be addressed in our study domain.^35^ This is illustrated in Figure 1 as a vicious cycle, depicting potential linkages of problematic social phenomena in supervision as described in our material.

We use these negative perceptions, instead of, for instance, positive or appreciative accounts, as they emerged abundantly and appear useful for pointing to three main entries for improvement of the supervisory relationship, as illustrated in Figure 1 and described in the following with supporting references.

### Entry 1: Supervision structure

Firstly, our study suggests that a supervision structure relying heavily on performance evaluations may induce substantial anxiety among providers and exacerbate the inherent power imbalance. The supervisory encounter in our material appeared to revolve around the performance indicators that supervisors mark, much like students may focus on subjects that are to be assessed in examinations.^36^ This may limit attention on the vastness of activities and work problems not included in evaluation checklists.^37^ Suggestions for structural changes were given in another paper,^14^ such as pre-announcing the date of evaluations and reducing the monthly frequency of evaluation visits to give supervisors time to offer formative encounters.

Another approach is to introduce a formal structure for feedback from providers to supervisors,^17^ particularly on aspects relating to building the supervisory relationship. Providers are the direct beneficiaries of supervision, and their feedback on supervision quality is essential to improving it. Similarly, as reported,^14^ HC managers share a responsibility for the outcome of supervision and may too benefit from a feedback channel.

Finally, in addition to the results presented our data had massive complaints about shortage of transport vehicles for supervision, high provider turnover, absenteeism and stressful work demands indicating precarious structural and organisational conditions that themselves may impair the supervision encounter and its supportive content. These structural challenges must be dealt with on policy and implementation levels.

### Entry 2: Interpersonal communication

Secondly, our material suggests that the quality of the interpersonal communication between external supervisors and providers is essential to the quality of the supervisory relationship and outcome of supervision. For instance, providers appear to benefit more from supervisors with a humble approach when correcting or advising, in spite of inherent power differentials.

External supervisors work in a conflict-prone environment, and the very process of evaluation is an occasion for disagreement, where controversy may be the rule rather than the exception.^37^ Anxiety may be expected and is to some extent beneficial in evaluation, yet in our data we find signs of so-called excessive evaluation anxiety, including conflict, avoidance, resistance, shame and anger,^17,18^ which may all inhibit performance. While this is unpleasant for providers, such environment may also wear down evaluators in their aspirations for objective evaluations and respectful professional relations. Over time, objectivity and the supervisory relationship may begin to come apart.^17^

In our data, several supervisors described providers’ feelings of anxiety and discomfort in reasonable proportion with the attention they received from providers, suggesting supervisors do not have problems interpreting providers’ emotional state. The better supervisors are prepared to take account of providers’ anxiety by involving them in the supervision process and purposes, acknowledging their feelings of discomfort,^38^ listening, providing useful feedback and encouraging them as part of critique, the more likely providers will be enabled to improve practice.^4,18^

Also, the more mindful supervisors are of the availability and use of power sources, the less is the risk of power misuse.^39^ This may help make the power relation as transparent as possible, whereas well-intended aims to simply shift the terminology of activities in favour of mentorship and support may conceal an inescapable power differential in the supervisory relationship.^14,40^ It may also promote the use of less formal power forms known from non-managerial supervision where supervisors hold no formal authority over the supervisee,^41^ but exert power through their professional expertise or the capacity to provide information perceived as useful by supervisees.^13^

### Entry 3: Support for weaknesses

Third, our data indicate that poor supervisory relationships often develop with providers perceived as insufficiently skilled. Providers performing poorly may experience low marks and negative feedback, which by itself does not seem to induce a change in practice. A notable reaction from some providers is to avoid supervision. A dilemma of supervision inequity may emerge, where those most in need of supervision are those most unlikely to benefit from it. This indicates that at least for a group of providers, rewards (which in their absence may be perceived as punishment) may be an ineffective power source and evoke fear and experiences of repeated failure. Our data imply that these providers often perceive the support system to oppose rather than meet their need for meaningful supervisory relations to guide their professional development, so that their competence and autonomous motivation remain unstrengthened.^9^ In response, any supervisee indeed has the power to reject the role as learner.^15^ Constructive attention towards providers with performance difficulties thus appears necessary for facilitating improved practice.

### Specific recommendations

The restrictive power of rewards is inherent to PBF. Applying promotive sources of power by engaging in non-judgemental interaction with providers to understand their needs and problems at work seems necessary to facilitate meaningful collaboration and sustainable improvements in practice.^21^ One approach would be to engage providers and supervisors in developing a supervisory contract to clarify roles, expectations and intentions.^16,18^ A supervisory contract should include ethical aspects concerning the supervisory relationship with reference to confidentiality, providers’ fear and welfare as well as proper communication.^18^ This may also address gender equality in the supervisory relationship because the majority of primary healthcare providers are female as opposed to the majority of supervisors being male, and also because female providers in one FGD shared experiences of supervisors using gender discriminating terms (such as saying ‘those women’ in a derogatory way). In the mixed FGD, male supervisors agreed that such terms were unacceptable. This together with the fact that other FGDs did not raise the issue of gender discrimination may indicate that such discrimination is the exception rather than the rule.

The supervisory role and responsibility must also be clear at a structural level. Role theory suggests individuals may act in ways they believe are socially expected from the roles they acquire.^42^ From this lens, it is important that required supervisory attitudes and behaviour are clear at policy and implementation level, so supervisors are informed and influenced from within their organisation rather than for example solely from traditional or cultural concepts of a supervisor.

Apart from a contract, we suggest to institutionalise open, provider-oriented conversations and mutual feedback channels in evaluative supervision visits. Although a dual role of evaluator and supporter may be difficult to fill, studies indicate it is possible for supervisors who develop an effective relationship with supervisees.^20,43^

As part of PBF, external and managerial supervision takes up substantial resources in Rwanda, which should be spent in ways that best help improve practice. We see a need for further studies on supervision, PBF and the problem of excessive evaluation anxiety in the Rwandan context. Questions to explore include how best to identify excessive evaluation anxiety, who are affected and how and the effect of strategies to prevent it. Further research is needed to explore reactions and optimal support of providers who experience multiple mediocre evaluations. Appropriate tools to measure external supervision quality should be locally validated and might serve as specific supervisor feedback. Interventional studies on supervision, PBF, alternative models and excessive evaluation anxiety should be in a controlled design with rigorous methods to quantify single intervention components using validated indicators.^3^ Finally, it has been suggested that medical doctors with a specialisation in primary healthcare, such as family physicians, play a role in the supervisory support of primary care providers in Africa.^29^ This could help ensure that supervision effectively addresses providers’ needs for developing their skills in response to the population’s needs for high-quality primary healthcare and should be a topic for further study.

### Limitations

Our study design does not simply allow transfer of results to other districts or HCs. It is theoretically possible that critique presented is unique to our sample. To explore transferability, we presented our findings on several occasions (latest in October 2016) to providers and supervisors from participating and non-participating facilities to request their critique and modifications. This confirmed and refined our findings and interpretation and brings confidence that results are still relevant and not representing isolated cases. Still, our data do not allow quantification of problems revealed, such as inappropriate use of power among supervisors and excessive evaluation anxiety among providers. They may be the exception rather than the rule, yet we are confident they are harmful and significant enough to require attention and action. Other studies would be needed to estimate their magnitude.

Social desirability bias is a risk to consider in any study that involves social interaction in generating data. We believe the low-moderator-involvement approach helped reduce this. In all FGDs, except the mixed FDG, participants already knew each other well. Participants frequently expressed their agreement and disagreement with others and related their points to something others said. Extreme positions were often modified or contested. Also, discussion topics were designed for open discussion allowing any polarity of views.^14^ We thus believe the data give a balanced and trustworthy picture of supervisors’ and providers’ positive and negative perceptions concerning the supervisory relationship.

## Conclusion

We found several signs of the so-called excessive evaluation anxiety among Rwandan primary healthcare providers, the scope of which should be further investigated. It appears to pose challenges for establishing a supervisory relationship appropriate for generating professional development among providers. A recommendation is for supervisors to adapt a style of communication and behaviour that acknowledges structural evaluation pressures and derived anxiety, and addresses it constructively. This may include the engagement in mutually developed supervisory contracts defining expected and intended roles and functions of all supervision forms and actors. Providers most in need of supervision to improve performance may be most unlikely to benefit from it. Particular efforts to encourage and support providers with performance difficulties are needed, as are structural changes to make the supervision scheme more responsive to providers’ context of challenges at individual facilities.

External supervision appeared driven by evaluation requirements of PBF with the potential to both improve and damage motivation of providers. The risk of harm calls for careful attention to factors that influence the supervisory relationship. The available forms of power should be recognised by supervisors, and used with caution. Rewards, which in their absence may be experienced as punishment, is a power form inherent to PBF in Rwanda, shaping the supervisory relationship. Promotive power sources that account for experienced and actual needs of providers appear decisive for desirable supervision outcomes.

While our study demonstrates that negative supervisory relationships may be detrimental to supervision outcomes and provider motivation thus aligning with reviews of supervision interventions in resource-constrained settings that did not confirm its effectiveness,^1,2,3,44^ this is not evidence against the assumption that constructive exchange between a provider and a supervisor has a crucial role to play for the continuous skill development and motivation of African primary care providers.
